# Influence of surface properties on the electrical conductivity of silicon nanomembranes

**DOI:** 10.1186/1556-276X-6-402

**Published:** 2011-05-31

**Authors:** Xiangfu Zhao, Shelley A Scott, Minghuang Huang, Weina Peng, Arnold M Kiefer, Frank S Flack, Donald E Savage, Max G Lagally

**Affiliations:** 1 University of Wisconsin-Madison, Madison WI 53706, USA; 2 School of Electronic Science and Engineering, Nanjing University, Nanjing 210093, China

## Abstract

Because of the large surface-to-volume ratio, the conductivity of semiconductor nanostructures is very sensitive to surface chemical and structural conditions. Two surface modifications, vacuum hydrogenation (VH) and hydrofluoric acid (HF) cleaning, of silicon nanomembranes (SiNMs) that nominally have the same effect, the hydrogen termination of the surface, are compared. The sheet resistance of the SiNMs, measured by the van der Pauw method, shows that HF etching produces at least an order of magnitude larger drop in sheet resistance than that caused by VH treatment, relative to the very high sheet resistance of samples terminated with native oxide. Re-oxidation rates after these treatments also differ. X-ray photoelectron spectroscopy measurements are consistent with the electrical-conductivity results. We pinpoint the likely cause of the differences.

PACS: 73.63.-b, 62.23.Kn, 73.40.Ty

## Introduction

Semiconductor nanomembranes (NMs), ultrathin layers of single-crystal semiconductor, can, because of the high surface-to-volume ratio, have electronic transport properties that are extremely sensitive to surface and interface conditions [1-3]. This surface sensitivity has a potential, so far not fully realized, for diverse applications, among others biological and chemical sensors [4-6], chemically gated transistors, or light-gated switches [7]. This sensitivity highlights the need for a greater understanding of the influence of chemical modifiers, even quite mundane ones, on the near-surface electronic properties and thus the charge transport properties of the semiconductor.

We consider here a model system, silicon nanomembranes (SiNMs). SiNMs can be either freestanding or bonded to a host substrate. In the simplest form, they are bonded to a SiO_2 _layer on a bulk Si substrate, so-called silicon on insulator (SOI). Very thin SiNMs under ambient conditions, i.e., terminated with oxide on all sides, are highly resistive because interface states trap most of the free charges. Consider, for example, a 10-nm thick crystalline sheet of Si with a nominal doping density of 10^15 ^cm^-3^. The sheet density of dopants in this thin membrane (10^9 ^cm^-2^) is much less than the typical trap density of even a high-quality Si/SiO_2 _interface (10^11 ^cm^-2 ^eV^-1^); thus, carrier depletion due to interface charge trapping renders the membrane effectively intrinsic. Consequently, any surface modification that significantly alters the trap density or provides free carriers by some other means (such as transfer doping via clean-surface states [2]) has a profound impact on the conductivity.

As an example of the extreme sensitivity of the conductivity of SiNMs to surface chemical condition, we describe in this paper the replacement of the surface oxide with a hydrogen termination [1,6,8-11] in two different ways. In one case, H-terminated SiNMs are prepared using surfaces cleaned in ultrahigh vacuum (UHV) that are dosed with pure hydrogen. The other approach uses the conventional HF wet etching of the oxide, which leaves the surface "H-terminated". Electrical transport properties are measured *via *the van der Pauw method in dry air (relative humidity <5%) and correlated with the surface chemical condition, determined from X-ray photoelectron spectroscopy (XPS). As suggested by prior work, the two treatments produce different surface chemistry [12-16]. The transport data demonstrate an extreme sensitivity of the conductivity to even trace surface chemical differences, a phenomenon totally absent in the bulk. The data lead us a step closer to an atomistic understanding of the complicated problem of the influence of surfaces on charge transport in semiconductor nanosystems.

## Background

The influence of the surface condition on silicon nanomembrane conductivity has been dramatically illustrated with scanning tunneling microscopy (STM) on Si(001). STM on clean bulk Si(001) produces high-quality images. Several attempts were made to image the surface of thin SOI(001) with STM, without success [17,18]. Considering the above estimate of charge carrier density when interface states are present, STM should not, in fact, be possible because STM requires a reasonably conducting sample. When the Si surface is carefully cleaned in ultrahigh vacuum (UHV), however, revealing the signature Si(001) 2 × 1 reconstruction and its associated surface state bands, STM images can be readily obtained with a quality similar to that of bulk samples. When the sample is not so carefully cleaned, and the 2 × 1 reconstruction is weak or absent, imaging is not possible. The mechanism for conduction for the clean, reconstructed surface is ascribed to an interaction between the surface bands produced by the 2 × 1 reconstruction and the "bulk bands" in the membrane. A thermally activated charge transfer between the bulk and surface bands, called "surface transfer doping," produces enough carriers to create high conductivity [3].

If the clean-surface bands are disrupted, the STM image quality rapidly degrades. So it is conjectured that incomplete removal of the oxide in UHV (oxide removal without destroying the template Si layer is quite a difficult problem for SOI), or any other kind of surface disorder, prevents formation of the 2 × 1 surface bands [3]. Additionally, the STM image degrades quickly if the clean surface of thin SOI (001) is exposed to H in UHV [19], while bulk Si(001) continues to be easily imaged [20]. Because the dimer-row-created surface band structure on clean Si is rapidly destroyed by H [19], the mechanism for conduction in thin SiNMs is eliminated.

The observation of H dosing of thin SOI(001) under UHV conditions producing a rapid drop in STM image quality (and therefore a presumed large reduction in conductivity) is, however, in contrast to recent electrical measurements of HF-treated membranes, which feature a dramatic increase in conductivity [1]. It is well known that HF etching of the oxide leaves a nominally H-terminated Si(001)surface, but with some residual F [12,13].

The STM measurements are only an indirect measure of conductivity; one can calculate for a given STM and given operating conditions what the limits on sheet conductance are to obtain a good image [2]. Whereas we are not able to duplicate here the exact conditions in UHV, because we at this stage cannot perform van der Pauw measurements in UHV, we prepare the vacuum H termination samples in a manner that permits dosing a nominally clean surface with clean H, as an intermediate step to the ultimate. We perform *ex situ *sheet resistance measurements and compare to H termination via HF etching for the same membrane thicknesses and sheet resistance measurement conditions.

## Experimental

P-type SOI(001) samples with nominal doping levels of 10^15 ^cm^-3 ^were patterned into 4 × 4 mm squares using photolithography and reactive-ion etching. Two different thicknesses of Si template layers (220 and 28 nm) were subjected to HF and vacuum H-dosing (VH) treatments. The choice of thicknesses is dictated by the earlier realization that 220 nm SiNMs behave close to bulk, while 28 nm SiMNs exhibit great surface sensitivity because the total dopant number is an order of magnitude smaller. The 28-nm samples were prepared with cycles of thermal oxidation and buffered oxide etching from the 220-nm commercial SOI wafers (Soitec, Peabody, MA, USA). From X-ray diffraction measurements of several samples diced from the same region of a wafer after thinning, we estimate that the thickness variation within each sample is less than 1 nm and the surface roughness, determined with atomic force microscopy, is typically 0.3 nm [1]. For HF treatment, samples were pre-cleaned with acetone and isopropyl alcohol, followed by a deionized (DI) water rinse, then dipped into 49% HF solution for 1 min, followed by a 1-min DI water rinse, and finally dried with flowing N_2_. For the VH treatment, samples were cleaned with Piranha (H_2_SO_4 _+ H_2_O_2_), AHP (H_2_O+NH_4_OH + H_2_O_2_), and dipped in 10% HF immediately prior to loading into the vacuum chamber. Once under vacuum (10^-9 ^torr), the sample was flash heated to 900°C to remove the *ex situ *H termination and obtain the 2 × 1 reconstruction (as determined by reflection high-energy electron diffraction). Silane in a H_2 _carrier gas was then flowed across the sample for 30 s while it was maintained at 580°C and for an additional 2 min after turning off the heat source. It is known that this procedure produces a H-terminated surface [21], and indeed, the samples were hydrophobic after removing from vacuum.

Indium point contacts were soldered to the sample immediately after terminating with hydrogen in both preparations. The sheet resistances were measured by the van der Pauw method [22] with a semiconductor parameter analyzer (Agilent 4156C, Agilent Technologies, Palo Alto, CA, USA) in dry air (relative humidity, RH < 5%) to restrict the influence of humidity because water vapor plays an important role in the conductivity of low-doped thin SOI [8]. The first sheet resistance measurement was always performed within 20 min of forming the H termination.

XPS measurements were performed with a PHI 5400 ESCA system (Physical Electronics, Division of Ulvac-PHI Chanhassen, MN USA) using Mg Kα X rays (1,254 eV). A larger take-off angle (45°) was chosen for survey spectra to obtain a stronger overall signal. A smaller take-off angle (15°), which brings more information from the surface, was selected for high-energy-resolution spectra to observe the change of the Si 2p core level peak in SiO_2 _to reveal the oxidation rate.

## Results and discussion

**Figure 1:**
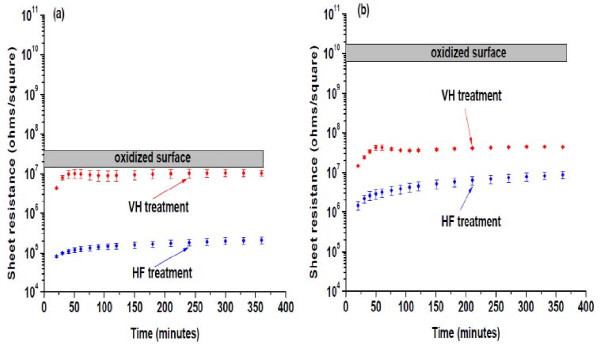
**The evolution of sheet resistance with time of Si nanomembranes in dry air**. After two surface modifications, VH and HF etching. (**a**) 220 nm. (**b**) 28 nm. The sheet resistance is lower for thicker NMs.

As mentioned above, the NM thicknesses chosen for these measurements provide total dopant sheet charge densities differing by about one order of magnitude for the same bulk dopant concentration. The thicker membrane should thus have lower resistance. Figure 1 shows a summary of the evolution of sheet resistance with time. The gray bar on top of each panel is the range of sheet resistance measured for the NM with an oxidized surface; no time dependence is measured nor expected. For oxide termination, the sheet resistance for the thinner NM is much higher, as predicted.

Figure 1a shows the evolution of the sheet resistance of 220-nm Si membranes in dry air for the two surface modifications. The data points are averages of several samples. The first data points were measured no more than 20 min after the surface modifications, as mentioned above. The HF treatment reduces the sheet resistance by more than two orders of magnitude compared to samples with the native-oxide termination (gray band). The sheet resistance increases slowly with time as the surface oxidizes gradually to form electrically active interface traps that deplete free carriers. This behavior is consistent with the results in Ref [1].

Figure 1a also shows that the behavior of the sheet resistance after treating with pure hydrogen under vacuum (VH) is quite different from the HF-treated case. The sheet resistance rapidly reaches a value only slightly below the sheet resistance of samples with a native-oxide surface. At early times, the sheet resistance may be as much as an order of magnitude lower than the oxide-termination values, but these measurements are less reliable. Such changes in conductivity would not be observable with bulk samples because the surface-to-volume ratio is much smaller for bulk samples, and the conducting paths through the bulk would drown out any changes due to surface modification.

Although the conductivity of SiNMs is increased, relative to the oxide termination, for both H surface modifications, the differences observed for the two suggest structurally or chemically different surfaces. In fact, HF treatment results in a sheet resistance even lower than the value calculated for a NM with bulk doping (10^15 ^cm^-3^) in the complete absence of interface traps, as has been shown earlier [3]. It has been suggested that the cause is residual species such as F and OH [1] and their chemical action with time [12,14,23].

Similar results are obtained for the 28-nm SiNMs, as shown in Figure 1b. Comparison of Figure 1a,1b shows that thinner Si membranes are more sensitive to the surface modifications (a much higher sheet resistance for the oxidized surface, as expected from discussions earlier, and a dramatic drop in sheet resistance with H termination). HF treatment causes about three orders of magnitude drop in the sheet resistance, while VH treatment produces over two orders of magnitude drop, relative to the oxidized NM surface. Interestingly, the post-treatment values are not as different from each other for the two NM thicknesses as are the values for the oxidized surface. The implication is that both H treatments reduce or eliminate the oxide-induced interface states and instead introduce somewhat differing states that do not trap as much charge.

**Figure 2:**
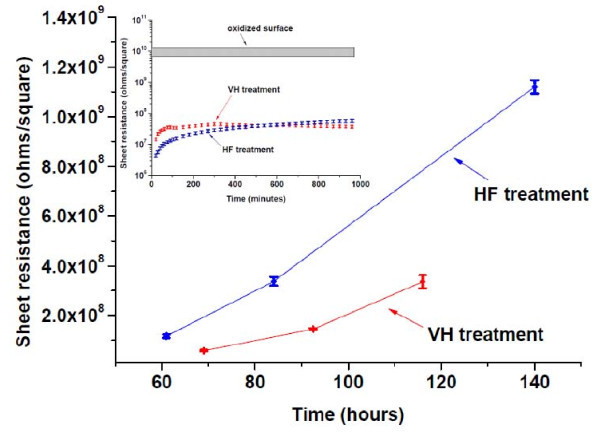
**Sheet resistance of 28-nm-thick Si membranes as a function of time**. After VH and HF treatments (linear scales). The inset shows the sheet resistance (log scale) for the first 16 h after treatment, showing the crossover point in sheet resistances.

Figure 1 also shows the initial evolution of the sheet resistance with time in an ambient (dry air) environment for both surface treatments. Figure 2 extends this time to 6 days for the thinner membrane. The inset (similar to Figure 1, but over a longer time) shows that for approximately the first hour, the sheet resistance of samples treated by VH increases faster than that of samples treated with HF, although the data in this regime are likely to be less reliable than for longer times. The sheet resistance of the HF-treated samples then increases more rapidly, crossing the sheet resistance of the VH-treated samples at approximately 8 h of exposure, after which time, the HF-treated samples become more resistive than those with the VH termination.

**Figure 3:**
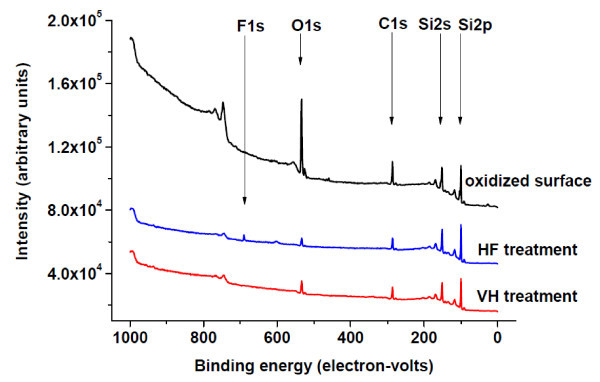
**XPS spectra of samples treated by the two surface modifications after 20-min exposure to air**. Take-off angle is 45°, pass energy is 89.45 eV. The unlabeled peak is the O Auger line.

It is known that after dosing with hydrogen under vacuum, the surface is purely H-terminated [16,21], while after HF treatment and DI water rinse, the surface is H-terminated with trace amounts of OH and F [12,14,23]. Figure 3 shows XPS spectra of 220-nm SiNMs treated by the two surface modifications after 20-min exposure to air. The samples treated with HF show a F 1s core level peak, confirming that the HF treatment leaves F ions on the surface [14], a feature that is absent in the VH-terminated sample. Comparison of the O1s peak for the H-terminated samples with the O1s peak from the sample with a native oxide confirms that the native oxide was removed after both H surface modifications.

It is attractive to regard the change in sheet resistance in terms of re-oxidation of the surface, i.e., the re-formation of oxide interface states from the condition of the surface produced by H termination. The simplest situation for the H-terminated surface would be complete passivation of surface states by H (including the "poisoning" of the clean-surface bands produced by the 2 × 1 reconstruction) [2,3]. If one adopts this model, then the evolution of sheet resistance plots the re-oxidation rate, and differences in re-oxidation rates of the NMs can be very sensitively inferred from the change of sheet resistance. There will then be a crossover point at 8 h where the degrees of oxidation of the two surface terminations switch. The VH termination appears then to be much more resistive to re-oxidation, i.e., it provides a much better surface chemical passivation than HF etching.

**Figure 4:**
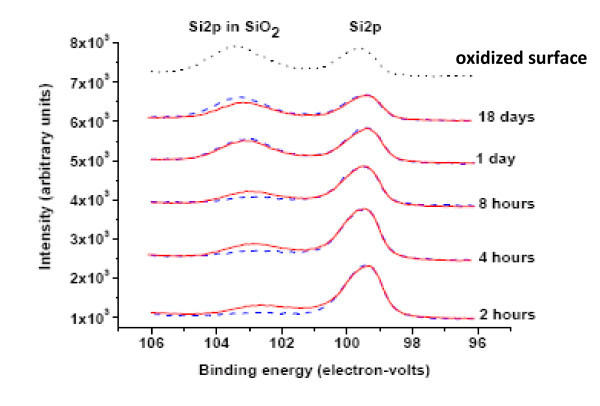
**Si 2p XPS core level peaks for Si and SiO_2_**. As a function of exposure time in air: red solid lines, blue dashed lines, and black dotted line correspond to samples treated by VH, HF, and with native-oxide surface, respectively. Take-off angle is 15°, pass energy is 35.75 eV. The top curve has been shifted by approximately 0.4 eV to facilitate comparison with the other curves. This shift is presumably due to surface charging.

There is XPS support for this conclusion. Figure 4 shows the Si 2p core level peak and corresponding chemically shifted peak due to presence of oxide as a function of exposure time in air. It is clear that with a 2-h exposure to air, the Si 2p core level peak in SiO_2 _of the sample treated by VH is stronger than that of sample treated by HF, indicating that the VH-terminated sample has a higher oxidation rate at the beginning. As the samples continue to oxidize, the magnitudes of the chemically shifted Si 2p core level peaks for each sample type become closer and eventually reach a similar peak height after an 8-h exposure to air. As oxidation continues past 1 day, the peak for the HF-treated sample becomes stronger than that for VH-treated sample and continues to increase over the 18 days of measurements, almost reaching the level of the sample with a native-oxide surface. Comparison of O1s peaks confirms increasing oxidation, relative to the initial stages (Figure 3), but at different rates, as predicted from the sheet resistance data. These observations are in excellent agreement with the results of the electrical measurements shown in Figures 1 and 2. From the inset in Figure 2, it is clear that within 1 day after surface modification, the sheet resistances of both sample types reach a similar value of around 3 × 10^7 ^ohms/square.

**Figure 5:**
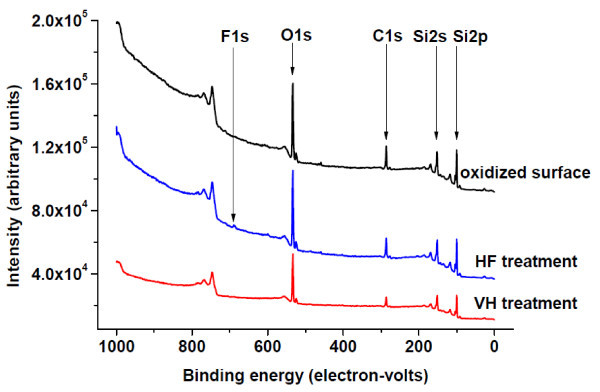
**XPS spectra of 220-nm SiNMs after 30 days exposure to air, compared to oxidized surface**. The F peak persists in the HF-treated SiNM. Take-off angle is 45°, pass energy is 89.45 eV.

There is, however, a caution. Low-doped (10^15 ^cm^-3^) p-type SiNMs with thicknesses of 220 nm or less show inversion of carrier type from p to n, even before any H termination treatments [10]. The conductivity that is measured is due to electrons, even though the material is nominally p-type [1]. This behavior can be due to trapped charges at the interfaces, whose effect becomes noticeable only for thin sheets, where the total number of bulk dopants is low. The sheet resistance returns to the initial (with native oxide) value within 30 days. Figure 5 shows XPS spectra after 30 days exposure to air. The small F 1s core level peak seen in Figure 3 can still be observed after a month of oxidation. The implication is that the trace surface F does not influence the ultimate sheet resistance. It is clear that the surface terminations with HF and vacuum hydrogenation differ and that the passivation induced by HF is much less effective than that induced by clean-H exposure of a vacuum-cleaned surface, but it appears that presence of F does not ultimately play the decisive role in the re-oxidation kinetics.

## Conclusions

Semiconductor nanomembranes offer an excellent platform to investigate small changes in surface chemical and structural differences, if appropriate electronic transport measurements are made. The reason is that the density of mobile charge in thin sheets or ribbons, and all Si nanowires, is so low at conventional doping levels that any small modification in the charge density, no matter from what source, significantly influences the conductivity. Such changes in conductivity would not be observable with bulk samples because the surface-to-volume ratio is much smaller for bulk samples and the conducting paths through the bulk would overwhelm any changes due to surface modification. Especially the oxidized surface, because of interface states, has a very high resistivity at conventional doping levels, and for that reason, studies employing Si nanowires invariably use close to degeneratively doped material.

To demonstrate this surface sensitivity, we investigated two surface modifications on thin SiNMs that are nominally identical but differ in trace amounts of adsorbed species, H termination via HF etching and H termination via exposure of a clean surface to H in an UHV system. The difference in these surfaces appears to be a trace adsorption of F, although we cannot be sure that H occupies the same surface sites in both situations. The results confirm the extreme sensitivity of the conductance of thin semiconductor sheets to chemical changes and demonstrate how thinness plays a role. In addition, we investigated the oxidation rate via sheet resistance measurements as a function of time. Within the assumption that oxide-produced interface states replace the H passivation, the sheet resistance sensitively tracks the coverage of oxygen on the surface, to the extent that one observes up to four orders of magnitude change in sheet resistance (depending on NM thickness, NM bulk doping, and nature of the surface modification) for the replacement of one monolayer of atoms. It is expected that this sensitivity can be exploited in future sensor applications using nanomembranes. It is also clear that nanomembranes provide an excellent vehicle for investigating interface states and defects that control the conductivity. In particular, electrical measurements in UHV (currently underway) will enable a more detailed understanding of the mechanisms of transport modulation via surface and interface modification in very thin semiconductors. That field of inquiry is still in its very early stages.

## Competing interests

The authors declare that they have no competing interests.

## Authors' contributions

XFZ made the measurements, with assistance from WP, SAS, AMK, and FSF. XFZ, SAS, WP, and MHH fabricated samples. All authors contributed to setting directions for the research and to discussions of the results and the manuscript. XFZ, SAS, and MGL participated in the preparation of the manuscript.
